# Effect of phytate on hypercalciuria secondary to bone resorption in patients with urinary stones: pilot study

**DOI:** 10.1007/s00240-022-01357-8

**Published:** 2022-09-10

**Authors:** Jordi Guimerà, Ana Martínez, Jose Luis Bauza, Pilar Sanchís, Enrique Pieras, Felix Grases

**Affiliations:** 1 Urology Department, Son Espases University Hospital, Carretera de Valldemossa, 79, 07120 Palma, Balearic Islands Spain; 2 University Health Science Research Institute (IUNICS), Science Department, Balearic Islands University, Palma, Spain

**Keywords:** Phytate, Hypercalciuria, Lithiasis, Bone resorption

## Abstract

The objective is to evaluate the effect of phytate supplements on calciuria in patients with urinary stones and elevated bone resorption. The secondary objective is to analyze the therapeutic effect of phytate based on measurements of serum markers of bone resorption. This is a controlled randomized study included patients according to predefined inclusion and exclusion criteria, and randomized them into two groups. Patients in the phytate group received a 380 mg capsule of calcium-magnesium InsP6 (Salvat Laboratories^®^) every 24 h for 3 months and patients in the control group received no treatment. All included patients were male or female, 18–65 years old, had hypercalciuria (> 250 mg/24 h), had a ß-Crosslaps level greater than 0.4 ng/mL, and had bone densitometry results indicative of osteopenia or osteoporosis in the femur and/or spine. At study onset, calciuria was 321 ± 52 mg/24 h in the phytate group and 305 ± 57 mg/24 h in the control group (*p* > 0.05). At 3 months, calciuria was significantly lower in the phytate group than the control group (226 ± 45 mg/24 h vs. 304 ± 58 mg/24 h, *p* < 0.05). At study onset, the mean ß-CrossLaps level was 1.25 ± 0.72 ng/mL in the phytate group and 0.57 ± 0.13 ng/mL in the control group (*p* < 0.05). However, at 3 months, the ß-CrossLaps level was significantly lower in the phytate group than in the control group (0.57 ± 0.13 ng/mL vs. 0.77 ± 0.42 ng/mL, *p* < 0.05). Phytate reduced calciuria in patients with hypercalciuria secondary to bone resorption. The ß-CrossLaps assay was effective for evaluating the efficacy of phytate on hypercalciuria during follow-up.

## Introduction

Kidney stone disease is a common condition, and it has estimated prevalence of 15% in the general population of the Balearic Islands [1]. Hypercalciuria is one of the main causes of urinary lithiasis, and accounts for an estimated 60% of all cases [2]. Furthermore, although urolithiasis and osteoporosis are two different pathological entities, the two diseases may share some similar pathogenesis pathway. Currently, multiple studies have linked hypercalciuria in renal lithiasis patients with elevated bone resorption [3–5]. In this sense, a prospective study that compared experimental group-subjects with calcium urolithiasis (*n* = 120) and control group without calcium urolithiasis (*n* = 120) concluded that osteoporosis is more prevalent in older patients and more pronounced in patients with calcium urolithiasis [3]. Another study with 22,575 patients with urolithiasis and 68,679 control patients of the Taiwan National Health Insurance Database (LHID 2000), concluded that urolithiasis significantly increases the subsequent osteoporosis rate [4]. All these facts indicate that osteoporosis increases the risk of hypercalciuria and urinary stones [5].

A 2011 study demonstrated the efficacy of bisphosphonate therapy in reducing hypercalciuria in patients with renal lithiasis and bone resorption [6]. Bisphosphonates are analogs of pyrophosphate with a high affinity for the hydroxyapatite of bone, especially in areas of rapid turnover and bone resorption [7, 8]. These drugs inhibit osteoclast activity, which causes a net increase in bone density, calcium deposition, and mineralization [9, 10]. Bisphosphonates such as alendronate, risedronate, and ibandronate have become useful in the treatment of hypercalciuria and hypercalcemia [6, 11–15].

Phytate (inositol hexakisphosphate, InsP6) is present in edible cereals, legumes, nuts, and seeds. Consequently, it is readily accessible to people consuming a balanced diet (1–2 g/day) rich in whole grains including legumes, whole cereals, and nuts. Although it is also found in all mammalian organs, tissues and fluids, the levels are low [16, 17] and are dependent upon exogenous supply either orally [16, 17] or topically [18, 19]. Urinary phytate can inhibit the crystallization of calcium phosphate and calcium oxalate lithiasis [20]. An animal study showed that phytate consumption inhibited osteoclast activity and bone resorption [21], and a subsequent clinical study demonstrated that phytate inhibited osteoporosis in menopausal women [22]. Phytate is a polyphosphate, so it can be considered an analog of bisphosphonates, so it has a similar behavior against osteoporosis [23–26]. In addition, the intake of phytate (InsP6), generates a set of derivatives (from InsP5 to InsP2), due to its dephosphorylation, which can explain both its efficacy against osteoporosis, and its inhibitory capacity of the crystallization of calcium salts [23].

The main objective of the present study is to evaluate the effects of a phytate supplement on hypercalciuria in patients with urinary stones and elevated bone resorption. The secondary objective is to analyze the relationships of the clinical characteristics of patients with the therapeutic effect of phytate based on measurements of serum markers of bone resorption.

## Materials and methods

All patients were selected according to predefined inclusion and exclusion criteria (see below) and randomized into the phytate group (one 380 mg capsule of calcium-magnesium InsP6 [Broken^®^ Salvat Laboratories] every 24 h for 3 months) or the control group (no treatment). The study protocol was approved by the Son Espases University Hospital Investigation Commission (Protocol #CI-474-20). All the patients included in this study were attended in the lithiasis unit from the urology department of Son Espases University Hospital.

The inclusion criteria were: male or female, age 18–65 years-old, hypercalciuria (> 250 mg/24 h), ß-Crosslaps level greater than 0.4 ng/mL, and bone densitometry results indicative of osteopenia or osteoporosis in the femur and/or spine. The exclusion criteria were: active treatment with bisphosphonates, active treatment with thiazides, active treatment with citrates, active treatment with glucocorticoids, use of calcium and/or vitamin D supplements, undergoing menopause, distal renal tubular acidosis, hyperparathyroidism, bone or connective tissue disease, or receipt of surgery of the spine or femur. All the patients enrolled in the study were recommended not to introduced high levels of phytate in their diets (edible cereals, legumes, nuts, and seeds. Randomized number table was the method for randomization.

The follow-up time was at least 3 months. The variables recorded at the beginning of the study were: age, sex, previous pathologies (arterial hypertension and/or diabetes mellitus), type of stone, laterality of stones, calciuria, ß-Crosslaps level and bone densitometry results. At 3 months after study onset, 24-h urinary calciuria and ß-CrossLaps level were reassessed. The serum ß -Crosslaps level was measured after diagnosing hypercalciuria (> 250 mg/24 h). Bone densitometry was performed after confirming the ß -CrossLaps level was above 0.4 ng/mL. Side effects of phytate treatment were recorded. ß -CrossLaps were measured with blood sample drawn after an overnight fast.

All patients received endoscopic surgery (ureteroscopy, flexible ureterorenoscopy, or percutaneous nephrolithotomy) or extracorporeal lithotripsy prior to study onset. The type of stone was defined using the Grases et al. [27] classification system, and stones were analyzed using stereoscopic microscopy, infrared spectrometry, and electron microscopy with energy dispersive X-ray microanalysis. Calciuria was measured in urine collected for 24 h.

Stones were classified as bilateral (both urinary tracts) or unilateral (one urinary tract). To determine the presence of urinary stones, abdominopelvic computed tomography (CT) was performed without contrast prior to the endoscopic surgery or extracorporeal lithotripsy.

T scores and bone mineral densities of the lumbar spine (L2–L4) and femoral neck were determined by dual X-ray absorptiometry (DXA; Norland Excell bone densitometer; MEC Osteoporosis Bone Densitometry, Minster, OH, USA). A single technician performed all measurements to avoid interobserver bias. Densitometric measurements were performed at the time of enrollment. Osteopenia was defined by a *t*-Score between − 1.0 and − 2.5, and osteoporosis by a *t*-Score of − 2.5 or less.

### Statistical analysis

Values are expressed as mean ± standard deviation (SD) or frequency (percentage). The normality of data distributions was analyzed using normality plots and tests. The baseline characteristics of patients in both groups were compared using a *t*-test for independent samples (quantitative data) and a Chi-square test or Fisher’s exact test (categorical data). Intragroup comparisons (before vs. after treatment) were performed using a paired-samples *t*-test. For intergroup comparisons, baseline values (before treatment) were compared using a *t*-test for independent samples and “after treatment values” were analyzed using ANCOVA with adjustment for baseline values. The percentage decreases of ß-Crosslaps level and 24-h urinary calcium level from before to after treatment were compared using a *t*-test for independent samples. All data were analyzed using IBM SPSS Statistics version 21. A two tailed *p*-value less than 0.05 was considered significant.

## Results

Twenty-three patients (10 women and 13 men) completed the clinical study (Table 1). Overall, the mean (± SD) age was 39 years (± 11). After randomization, there were 12 subjects in the phytate group and 11 in the control group. All stones were calcium oxalate dihydrate (COD), hydroxyapatite (HAP), brushite (B), or a mixture of these components.

**Table 1:** Baseline characteristics of patients for both groups

	Phytate group (*n* = 12)	Non-phytate group (*n* = 11)	*p*-value
Age (years)	35.6 ± 12.7	43.6 ± 7.2	0.075
Gender (male)	7 (58.3%)	6 (54.5%)	1.000
Hypertension	1 (8.3%)	0 (0%)	1.000
*Type of calculi*			
B + HAP	0 (0.0%)	1 (9.1%)	0.130
B + HAP + COD	0 (0.0%)	1 (9.1%)	
COD	3 (25.0%)	4 (36.4%)	
COD + HAP	9 (75.0%)	3 (27.3%)	
HAP	0 (0%)	2 (18.2%)	
Bilaterality of renal lithiasis	12 (100%)	10 (90.9%)	0.478
*Lumbdar Spine*			
Normal	8 (66.7%)	4 (36.4%)	0.125
Osteopenia	1 (8.3%)	5 (45.5%)	
Osteoporosis	3 (25.0%)	2 (18.2%)	
*Femur*			
Normal	0 (0.0%)	2 (18.2%)	0.300
Osteopenia	9 (75.0%)	7 (63.6%)	
Osteoporosis	3 (25.0%)	2 (18.2%)	

Values are expressed as mean ± SD or frequency (percentage)

*B* Brushite, *HAP* hydroxyapatite, *COD* calcium oxalate dihydrate

**Fig. 1:**
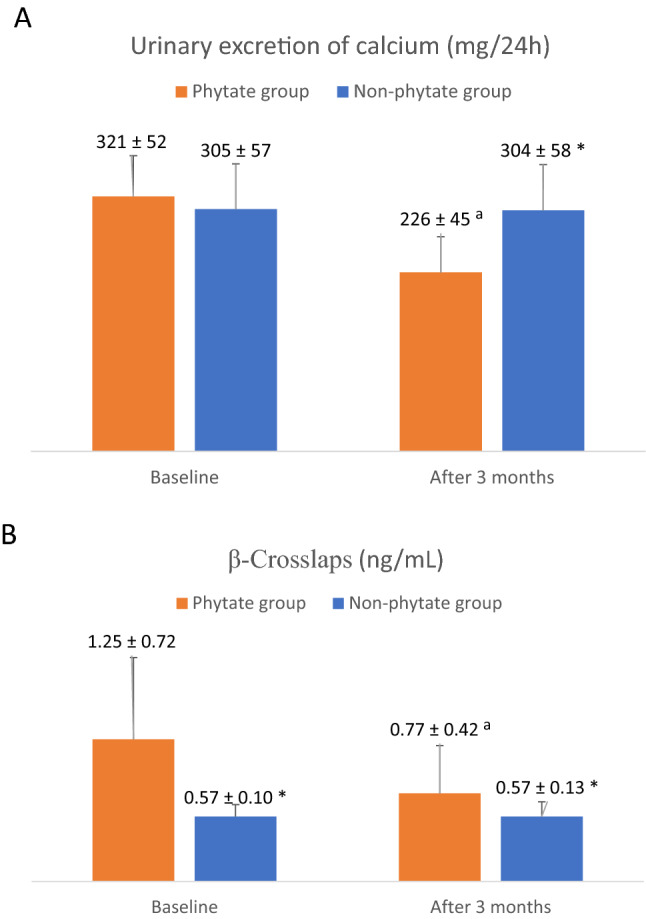
Urinary excretion of calcium (**A**) and beta crosslaps (**B**) values before and after 3 months of treatment for both groups. ^a^*p*-value < 0.05 vs. baseline corresponding value. *p-value < 0.05 vs. corresponding value of phytate group

At study onset, the 24 h urinary calcium level was 321 (± 52) mg in the phytate group and 305 (± 57) mg in the control group (*p* > 0.05; Fig. 1A). After 3 months the 24 h urinary calcium level was significantly lower in the phytate group (226 ± 45 vs. 304 ± 58 mg, *p* < 0.05).

**Fig. 2:**
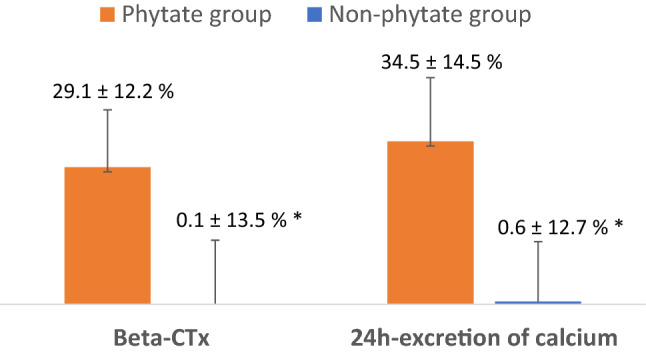
Percentage of decrease of beta-CTx and 24-h excretion calcium values before and after treatment for both groups. Percentage was calculated as [(value at baseline − value after 3 months)/value at baseline × 100]. *p-value < 0.05 vs. corresponding value of phytate group

At study onset, the ß -CrossLaps level was significantly greater in the phytate group (1.25 ± 0.72 vs. 0.57 ± 0.13 ng/mL, *p* < 0.05; Fig. 1B). However, after 3 months, the ß -CrossLaps level declined significantly more in the phytate group than in the control group (1.25 ± 0.72 to 0.77 ± 0.42 vs. 0.57 ± 0.10 to 0.57 ± 0.13 mg/mL, *p* < 0.05). Thus, the phytate group had a 29.1% (± 12.2) decrease in the ß-CrossLaps level and a 34.5% (± 14.5) decrease in calciuria, but the control group only had negligible changes in these values (Fig. 2).

## Discussion

Hypercalciuria is considered the most common risk factor for urinary stones [2]. It is considered hypercalciuria as being caused by increased intestinal absorption of calcium, renal calcium leakage due to a kidney disease, disruption of calcium resorption (as in hyperparathyroidism), idiopathic hypercalciuria or renal phosphate leakage. We consider an additional type of idiopathic hypercalciuria secondary to idiopathic bone resorption, in the absence of: hyperparathyroidism, distal renal tubular acidosis, connective tissue/bone diseases, menopause, treatment with corticosteroids and hypervitaminosis D. The percentage of hypercalciuria secondary to idiopathic bone resorption is not known.

Is well stablished that potassium citrate is the treatment for idiopathic hypercalciuria if the urinary pH is below 5.5. A thiazide-type diuretic could also be administered in some cases. We examined the effect of a phytate supplement, as an inhibitor of bone resorption and a modulator of hypercalciuria, in patients with kidney stones. To our knowledge, this is the first study to examine the effect of phytate as a treatment for hypercalciuria secondary to bone resorption in patients with urolithiasis.

It is known that the patients believe that osteoporosis drugs have side effects and are minimially effective for treating the osteoporosis. It is believed that nutrition, exercise, and diet are effective for treatment of osteoporosis. The number of prescriptions for bisphosphonates decreased by 50% from 2008 to 2012 [28]. Phytate (InsP6), which is present in edible seeds, legumes, nuts, and whole cereals, inhibits the crystallization of calcium phosphate and calcium oxalate [29, 30], and also inhibits bone resorption [21–23]. After 3 months, our phytate group had a greater decrease in calciuria than the control group. In fact, the control group had no change in hypercalciuria. This demonstrated that patients with hypercalciuria secondary to idiopathic bone resorption who used a phytate supplement had decreased calciuria. In addition, because phytate acts as an inhibitor of urinary crystallization, it may also benefit these patients by decreasing the risk of lithiasis. There is evidence that oral phytate inhibits pathological calcifications and bone resorption, and leads to the appearance of the different InsPs in the blood and urine due to the hydrolysis of InsP6 into InsP5, InsP4, InsP3, InsP2 [23].

Previous research established relationships of osteoporosis, hypercalciuria, and urinary stones [31–33], although the limited research in this area has led to under-treatment and under-diagnosis of this pathology. Arrabal et al. [6] identified relationships in the levels of serum markers of bone resorption, loss of bone mass, and urinary lithiasis. In this study, ß -CrossLaps have been used to make the decision to perform bone densitometry on patients, a value > 0.4 has been used to perform the densitometry based on the study by Arrabal et al. [6]. Okabe et al. [34] demonstrate that ß -CrossLaps are one of the most useful serum makers for predicting BMD and the response to antiresorptive treatment.

Despite our small sample size, we observed a statistically significant decrease in the ß-CrossLaps level in the phytate group, but not in the control group. This implies that ß-CrossLaps could be used as a surrogate variable for hypercalciuria during patient follow-up.

Our analysis of the types of kidney stones indicated that our patients had COD, HAP, B, or mixed stones. These types of stones mainly form in the presence of high concentrations of calcium, as indicated by hypercalciuria [27, 35]. COD, HAP, B, or mixed urinary stones account for 50–53% of all urinary stones [27], and hypercalciuria is the most common urinary disorder in patients with urinary stones (50–60%) [2]. Stone bilaterality is a well-known risk factor for stone recurrence [36]. Notably, 22 of our 23 subjects (96%) had bilateral stones. The patients of our study with the following characteristics such as young age, bilateral stone and stone composition have high recurrence and is often under-diagnosed and under-treated [37]. Early identification of patients with these characteristics can allow early treatment with phytate, which inhibits crystallization, modulates calciuria, and restores bone mass. More studies are required to determine the prevalence of patients with hypercalciuria secondary to idiopathic bone resorption.

Analysis of bone loss in our patients indicated that 47.8% of them had exclusive involvement of the femur, 8.7% had exclusive involvement of the spine, and the others were involved of both bones. Previous research reported that loss of bone mass was constant over time in both sexes for cortical and trabecular bone [32], although the onset of menopause led to greater loss of trabecular bone [38]. We are uncertain why our patients had greater involvement of the cortical bone. A longer follow-up (e.g., 24 months) with continuous use of phytate and additional bone densitometry measurements may clarify this issue.

The mean age of our patients was 39 years. However, if we consider that the sample size was small and only 3 patients were older than 50 years, our patients can be considered quite young. Identification of hypercalciuria in these young patients is important, because they can develop multiple recurrences of renal lithiasis and progressive loss of bone mass as they get older. Although we believe that phytate is likely to reduce the incidence of stone recurrence and progression, our short follow-up time cannot determine this outcome.

The major limitations of this study were the small sample size and the short follow-up time. A larger sample size would allow us to confirm the predominance of cortical bone involvement in patients with hypercalciuria secondary to bone resorption. A longer follow-up time with additional bone densitometry measurements would allow us to better assess the effect of phytate on bone remodeling. Dietary consumption of phytate before and during the intervention was not quantified, the consumption of phytate could modify the results of our study.

## Conclusion

Our results indicated that phytate reduced calciuria in patients with hypercalciuria and incrised bone resorption. The ß-CrossLaps level can be used during follow-up to evaluate the efficacy of phytate in reducing hypercalciuria.
